# The Novel 2.3.4.4b H5N6 Highly Pathogenic Avian Influenza Viruses Isolated From Wild Birds in 2023 Posing a Potential Risk to Human Health

**DOI:** 10.1155/2024/4900097

**Published:** 2024-10-22

**Authors:** Yuting Xu, Jie Hu, Chenyao Zhao, Yue Yuan, Zijing Gao, Zhenghuan Wang, Kirill Sharshov, Guimei He

**Affiliations:** ^1^School of Life Sciences, East China Normal University, Shanghai, China; ^2^Shanghai Forestry Station, Shanghai, China; ^3^Shanghai Chongming Dongtan Nature Reserve Administration Center, Shanghai, China; ^4^Research Institute of Virology, Federal Research Center for Fundamental and Translational Medicine, Siberian Branch, Russian Academy of Sciences Novosibirsk, Russia; ^5^Institute of Eco-Chongming (IEC), East China Normal University, Shanghai, China; ^6^Shanghai Institute of Wildlife Epidemics, East China Normal University, Shanghai, China

**Keywords:** 2.3.4.4b H5, H5N6, reassortment, wild birds

## Abstract

The highly pathogenic avian influenza 2.3.4.4b H5 viruses have been a cause for concern recently, as they have been responsible for continuous outbreaks since 2021. In China, the H5N6 subtype has been predominantly circulating in domestic poultry but has rarely been detected in wild birds over the past 3 years. In December 2023, novel reassortant 2.3.4.4b H5N6 viruses were resurgent in wild birds and domestic ducks in Eastern Asia. The viruses were reassorted with those of currently prevalent 2.3.4.4b H5N1 viruses of wild bird origin worldwide, as well as the H5N6 viruses that caused human infections in 2022 and low pathogenic avian influenza viruses, such as the H9N2 virus, which also contributed internal gene to the novel H5N6 viruses. Based on the phylogenetic analyses, we inferred that this recombination process occurred in migratory breeding sites in early 2023. Given the rapid transmission and high mutation capacity of currently circulating H5N1 viruses, as well as the strong pathogenicity of H5N6 viruses to humans, the novel recombinant viruses may continue to evolve and pose new threats to human health. Therefore, continuous surveillance of H5N6 viruses in wild birds and domestic poultry should be strengthened.

## 1. Introduction

The ongoing outbreaks of highly pathogenic avian influenza (HPAI) viruses, specifically clade 2.3.4.4b, have been attributed to infections and mortalities in birds, mammals, and even humans across all continents [1]. The recent identification of clade 2.3.4.4b HAPI H5N1 viruses in US dairy cows, milk, and farm workers has raised concerns regarding the pandemic potential, once again highlighting the threats posed to public health [2].

In addition to extensive transmissibility, the 2.3.4.4b clade HPAI H5 viruses demonstrated a remarkable capacity for rapid evolution through reassortment with other subtypes of influenza virus, resulting in the emergence of H5N1, H5N2, H5N3, H5N4, H5N5, and H5N6 viruses [3]. Since 2013, the HPAI H5N6 viruses have occurred in Asia [4] and subsequently widely circulated in poultry and wild birds [5]. In China, the H5N6 subtype has replaced H5N1 as one of the dominant subtypes of highly pathogenic avian influenza virus (HPAIV) in domestic poultry [6]. Of particular concern is the significant increase in human and mammal infections caused by the 2.3.4.4b clade H5N6 virus since 2021, including farm canines, swine, and felines [7]. A total of 63 cases of H5N6 infection were documented among humans, resulting in 27 fatalities over the last 3 years [8]. Therefore, the H5N6 viruses may pose a pandemic potential for public health.

Wild waterfowl as a natural reservoir of AIVs play a significant role in the global dissemination of HPAIVs through long-distance migration. Eastern China, as a critical region for bird migration, poses a potential risk for HPAIV transmission through migratory birds. In 2016, H5N6 viruses were circulating among migratory waterfowl in eastern China [9]. Subsequently, the HPAIV H5N6 viruses were gradually replaced by the H5N8 and H5N1 viruses in wild birds. In December 2023, we identified a 2.3.4.4b clade H5N6 virus from an apparently healthy spot-billed duck in Shanghai, eastern China, with all genes phylogenetically distinct from those of viruses detected in wild birds and domestic poultry over the past 3 years.

## 2. Materials and Methods

### 2.1. Ethics Statement

Wild birds were captured and sampled with permission of and approval by the Shanghai Forestry Bureau (2023 [54]). After sampling, all birds were released. The experiments involving viruses were conducted in a biosafety level 2 (BSL-2) laboratory at East China Normal University.

### 2.2. Sample Collection, Viral Ribonucleic Acid (RNA) Isolation, and Polymerase Chain Reaction (PCR) Identification

Throughout 2023, a total of 2463 samples from 1636 wild bird species were collected in Nanhui Dongtan wetland (30°51′ to 31°06′ N, 121°50′ to 121°51′ E) and Chongming Dongtan wetland (31°25′−31°38′ N, 121°53′−122°04′ E), Shanghai, China. These samples comprised 1611 swab samples, 802 fecal samples, and 50 tissue samples. An experienced ornithologist identified bird species after capture, and their species, state of health, and sex (if available) were recorded. Viral RNAs were extracted using the MagMAX Pathogen RNA/DNA Kit (Thermo Fisher Scientific, Waltham, MA, USA) on a MagMAX Express-96 Deep Well Magnetic Particle Processor (Thermo Fisher Scientific, Waltham, MA, USA) according to the manufacturer's protocol. Influenza A viruses were tested using real-time reverse transcription PCR (rRT-PCR) assay targeting the matrix gene on a 7500 Real-Time PCR instrument (Thermo Fisher Scientific, Waltham, MA, USA). The viral RNAs of positive samples were transcribed into complementary DNA (cDNA) using the Uni12 primer (5′-AGC AAA AGC AGG-3′) and a PrimeScript II First Strand cDNA Synthesis Kit (Takara, Tokyo, Japan). Consequently, the H5N6 AIV was detected from a spot-billed duck on December 5, 2023, in Nanhui Dongtan wetland. Then the eight gene fragments of the H5N6 isolates were sequenced using universal primers [10]. The genome sequences have been deposited in the Global Initiative on Sharing All Influenza Data (GISAID) database (https://gisaid.org/).

### 2.3. Phylogenetic and Bayesian Evolutionary Analysis

We downloaded the relevant reference sequences of eight gene fragments from the GISAID database. All gene sequences were aligned using MAFFT v7.520 [11]. Phylogenetic trees of eight genes of the H5N6 virus were constructed using the maximum likelihood (ML) trees in IQ-tree v2.2.2.6 (http://www.iqtree.org/) [12] with 1000 bootstrap analyses. Divergence times and evolutionary rates were estimated using an uncorrelated relaxed clock model under a Bayesian framework using Markov chain Monte Carlo (MCMC) sampling in BEAST v.1.10.4 [13]. The MCMC chain length was set with 200 million states with 10% burn-in. Tracer v1.7.2 (http://tree.bio.ed.ac.uk/software/tracer/) was used to calculate the effective sample size (ESS), which should be over 200. To summarize the information of sampled trees, we used the TreeAnnotator v1.10.4 tool and ultimately produced a summary tree that can be displayed using FigTree v1.4.4 (http://tree.bio.ed.ac.uk/software/figtree/).

## 3. Results and Discussion

During our routine surveillance of AIVs in 2023, the total prevalence of AIVs was 5.87% (*n* = 96, 95% confidence interval [CI], 4.80%−7.15%), including H2N3, H3N8, H4N2, H5N1, H5N6, H6N2, H6N5, and H11N9. Among these, a novel H5N6 virus was detected in cloacal and oropharyngeal swabs collected from an apparently healthy spot-billed duck (*Anas poecilorhyncha*) in the winter of 2023 in Shanghai, China. The virus was designated as A/spot-billed duck/Shanghai/NH23997/2023 (H5N6) (referred to as 23997-H5N6). All the full-length genome sequences were analyzed for further analysis. These sequences have been deposited in the GISAID database (https://gisaid.org/) under accession numbers EPI2932843, EPI2932902, and EPI2932983-EPI2932988. A GISAID Basic Local Alignment Search Tool (BLAST) search (https://platform.epicov.org/epi3/frontend#5413a9) revealed that the eight gene segments of the 23997-H5N6 virus shared 99.55%–100% nucleotide identity with those of the H5N6 viruses isolated from peregrine falcon (*Falco peregrinus*) in Japan, mandarin duck (*Aix galericulata*), and domestic ducks in Korea in December 2023 [14], indicating the five H5N6 viruses might originate from a common ancestral virus and were subjected to analyze together (collectively referred to as 2023-H5N6-like viruses).

**Figure 1:**
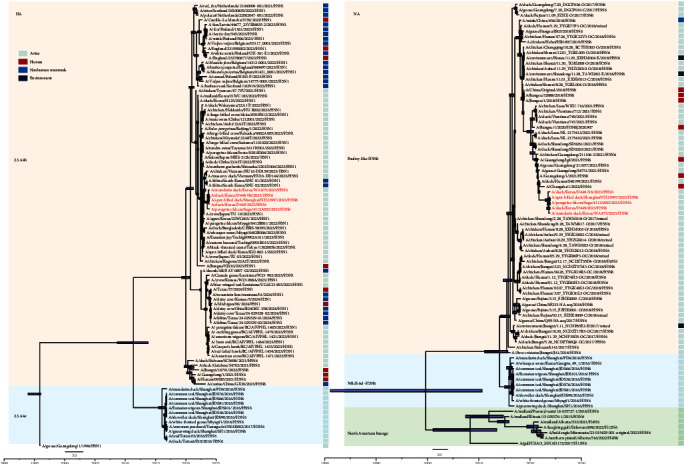
Maximum clade credibility time-scaled phylogenetic trees of the hemagglutinin gene and neuraminidase gene of 2023-H5N6-like viruses. The 2023-H5N6-like viruses were shown in red. On the right are the host annotations with different colors.

**Figure 2:**
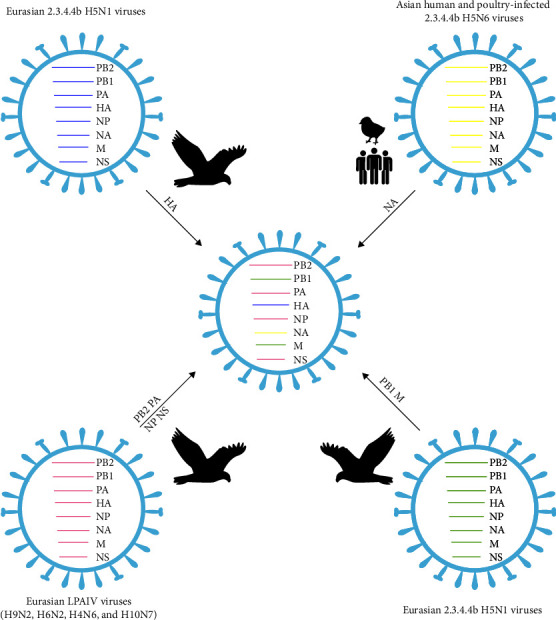
Schematic of the eight viral gene segments of 2023-H5N6-like viruses. Genes are colored according to the phylogenetic tree analyses and classification. LPAIV, low pathogenic avian influenza virus.

The phylogenetic analyses of the whole genomic sequences of 2023-H5N6-like viruses were performed. The HA genes of 2023-H5N6-like viruses were clustered into the 2.3.4.4b clade in the maximum clade credibility (MCC) trees and further grouped with Eurasian H5N1 viruses from 2021 to 2023, which have caused massive infections in humans and other mammals, including foxes, minks, and seals (Figure 1). The M and PB1 genes of 2023-H5N6-like were also closely related to those of wild bird-originated 2.3.4.4b H5N1 viruses in Eurasian regions (Figure S1). The NA genes were grouped with the human-infected H5N6 viruses, as well as the prevalent H5N6 viruses in domestic poultry in 2021–2022 (Figure 1). However, the long branch of the clade of 2023-H5N6-like viruses in the NA phylogenetic trees suggested that they may have undergone multiple mutations or reassortments (Figure 1). The other four internal genes were clustered into Eurasian lowly pathogenic AIVs (Figure S1). Therefore, the 2023-H5N6-like viruses were novel reassortants involving wild bird-origin 2.3.4.4b H5N1 viruses and human and domestic poultry H5N6 viruses in 2021–2022, as well as other internal genes derived from Eurasia LPAIVs, such as H9N2 viruses (Figure 2). The recombination events of the 2023-H5N6-like viruses may have occurred in early 2023 at breeding or wintering sites according to the estimated time to the most recent common ancestor (tMRCA) (Table S1). Given the shared migratory flyway among Eastern China, Japan, and Korea, it is reasonable to hypothesize that the novel recombinant H5N6 viruses in late autumn 2023 can be attributed to migratory birds from breeding sites.

The concurrent detection of this novel 2.3.4.4b H5N6 viruses in Eastern Asia implies that the viruses seemed to be well-adapted in migratory wild birds, thereby raising concerns regarding their potential ability to asymptomatically spread viral pathogens over long distances. On the one hand, the cross-regional transmission of the virus is facilitated by migratory movements of wild birds, while on the other hand, the cross-species transmission is also increased through poultry being exposed to viruses due to their shared habitats with wild birds. Furthermore, the novel 2.3.4.4b H5N6 viruses can infect domestic duck in Korea, and our findings indicate that the 23997-H5N6 has undergone multiple mutations at antigenic sites B (185, 192, and 193) and C (276) when compared to the vaccine strain Re-14 [15, 16] (Table S2), highlighting the potential risk of an outbreak in poultry and the subsequent spillover to humans due to possible inadequacy of current commercial vaccines in providing complete protection.

The prevalence of H5N1 AIVs is currently increasing in Europe and America, and they have infected multiple wild mammals and farm animals [17]. Furthermore, the H5N6 virus is widespread among domestic poultry in China, and human infection with the H5N6 viruses has shown even higher mortality rates than those caused by the H5N1 viruses based on current clinical statistics [18]. It is noteworthy that a recent rise in the frequency of 2.3.4.4b clade H5N1 and H5N6 cases has been observed. The cocirculation of H5N6 and H5N1 viruses in migratory birds may accelerate the evolution of novel variants, increasing their ability for recombination, pathogenicity, and dissemination. Eastern Asia has been a host spot for the emergence of novel genetic reassortments. Therefore, increased surveillance is needed in these regions to further elucidate the origin and evolution of these viruses, thereby providing crucial early warning that could potentially prevent an influenza epidemic in humans.

## 4. Conclusions

In this study, we identified a novel influenza A (H5N6) virus in wild waterfowl in Shanghai in 2023. These findings provide evidence of H5N6 virus recombination, highlighting the role of wild waterfowl for virus cross-region and cross-species transmission. We also demonstrated that this recombinant H5N6 virus may acquire the escalated transmission of 2.3.4.4b H5N1 viruses and the high pathogenicity of H5N6 viruses in domestic poultry and humans, pausing great threats to the poultry industry and human health. Thus, we called for continued attention and monitoring of this novel H5N6 virus worldwide.

## Data Availability

All sequence information for this article is available on the GISAID (https://gisaid.org/) database under the serial numbers EPI2932843, EPI2932902, and EPI2932983-EPI2932988.

## Supporting Information

Supporting Information Table S1. The highest nucleotide homology and tMRCA of the whole genome for the 2023-H5N6-like viruses. Table S2. Mutations detected in the 23997-H5N6 virus. Figure S1. Maximum likelihood trees of six internal genes: (a) PB2, (b) PB1, (c) PA, (d) NP, (e) M, and (f) NS of the 2023-H5N6-like viruses (red) isolated in 2023.
