# Supplementary material for: The Novel 2.3.4.4b H5N6 Highly Pathogenic Avian Influenza Viruses Isolated From Wild Birds in 2023 Posing a Potential Risk to Human Health
Source: Transbound Emerg Dis. 2024 Oct 22;2024:4900097. doi: 10.1155/2024/4900097 (PMC12016695; doi:10.1155/2024/4900097)
Supplement: Supporting Information — Table S1. The highest nucleotide homology and tMRCA of the whole genome for the 2023-H5N6-like viruses. Table S2. Mutations detected in the 23997-H5N6 virus. Figure S1. Maximum likelihood trees of six internal genes: (a) PB2, (b) PB1, (c) PA, (d) NP, (e) M, and (f) NS of the 2023-H5N6-like viruses (red) isolated in 2023. [file 4900097.f1.docx]

**Supplementary Materials**

**Table S1.** The highest nucleotide homology and tMRCA of the whole genome for the 2023-H5N6-like viruses

| Gene | The most homologous strains | Identity(%) | tMRCA | Lower 95% tMRCA | Upper 95% tMRCA |
| --- | --- | --- | --- | --- | --- |
| PB2 | A/environment/chongqing/1795/2023 (A/H9N2) _like | 99.56 | 2023/5/21 | 2022/12/31 | 2023/9/26 |
| PB1 | A/large-billed crow/Kanagawa/1403C006/2023 (A/H5N1)_like | 99.68 | 2023/5/7 | 2023/1/1 | 2023/8/31 |
| PA | A/common teal/Amur region/92b/2020 (A/H6N2)_like | 99.62 | 2023/7/23 | 2023/2/14 | 2023/11/10 |
| HA | A/duck/Chiba/22A3T/2022 (A/H5N1) _like | 99.62 | 2023/6/16 | 2023/3/9 | 2023/9/16 |
| NP | A/gadwall/Novosibirsk region/3407k/2020 (A/H4N6)_like | 98.71 | 2023/1/13 | 2022/4/18 | 2023/8/21 |
| NA | A/Changsha/1/2022 (A/H5N6)_like | 98.46 | 2023/6/14 | 2022/12/22 | 2023/10/30 |
| M | A/Mallard/Korea/21WS41-5/2022 (A/H5N1)_like | 99.78 | 2023/9/5 | 2023/6/20 | 2023/11/12 |
| NS | A/bean goose/Korea/KNU-10/2022 (A/H10N7)_like | 99.74 | 2023/4/12 | 2022/9/21 | 2023/10/2 |

**Table S2.** Mutations detected in the 23997-H5N6 virus

| Gene | Site | | 23997-H5N6 | Re-14 | Phenotype information |
| --- | --- | --- | --- | --- | --- |
| HA | SiteB | 158 | D | N | The mutations at antigenic sites compared to the vaccine strain Re-14 |
|  |  | 160 | A | A |  |
|  |  | 185 | P | S |  |
|  |  | 192 | T | I |  |
|  |  | 193 | K | N |  |
|  | SiteC | 276 | H | D |  |
| NA | 54-72 | | deletion | / | Enhanced receptor binding activity |
| PB2 | K389R | | R | / | Increased polymerase activity |
|  | V598T | | T | / |  |
|  | T339K | | K | / |  |
|  | R477G | | G | / |  |
|  | I495V | | V | / |  |
|  | K627E | | E | / |  |
| PA | S515T | | T | / | Increased polymerase activity |
| NP | A184K | | K | / | Increased virulence in chickens or ducks; Enhanced IFN response |
| M | I43M | | M | / | Increased virulence in mice |
|  | T215A | | A | / |  |
| NS | C138F | | F | / | Enhanced replication in mammalian cells |
|  | V149A | | A | / | Increased virulence in chickens or ducks |
|  | L103F | | F | / | Increased virulence in mice |
|  | I106M | | M | / | Increased virulence in mice |
|  | K55E | | E | / | Enhanced replication in mammalian cells |
|  | K66E | | E | / | Enhanced replication in mammalian cells |


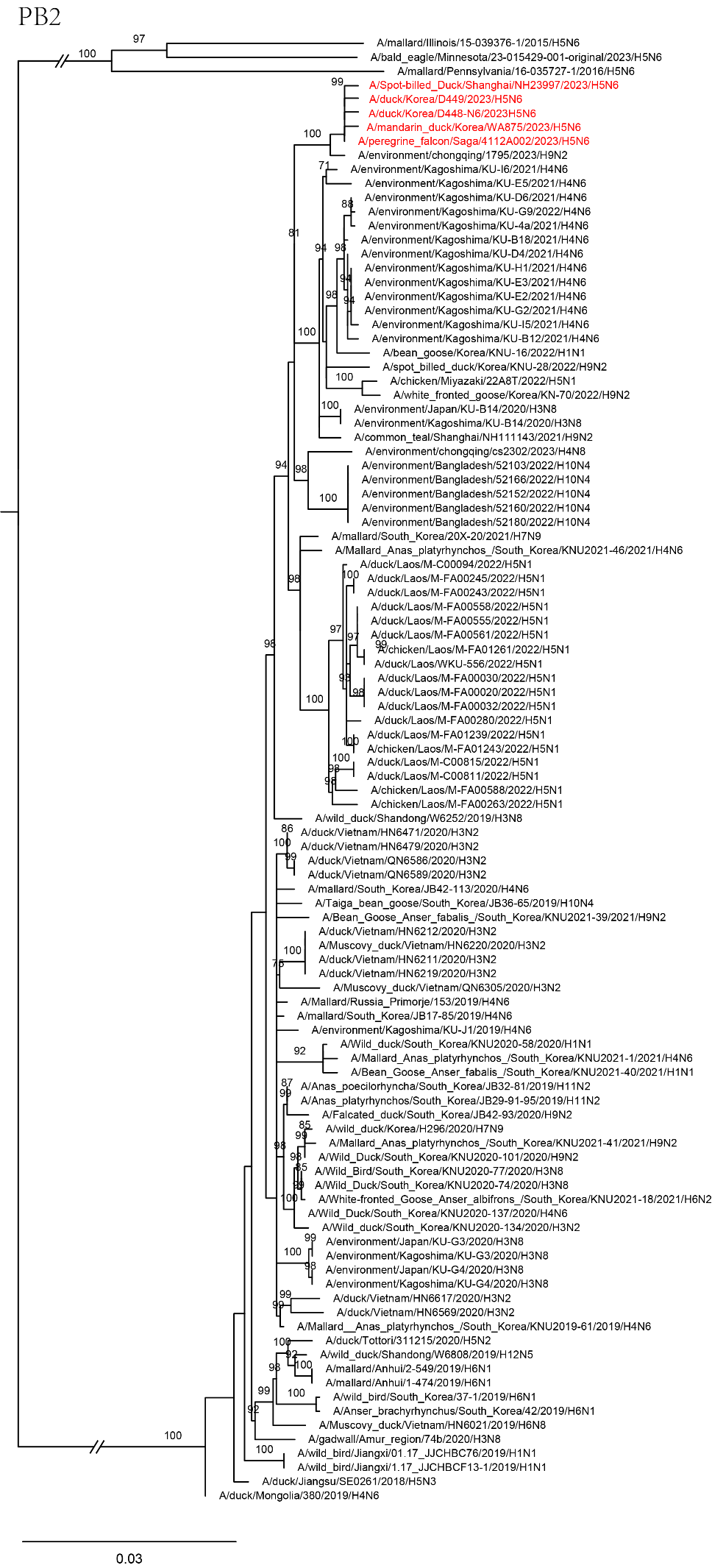


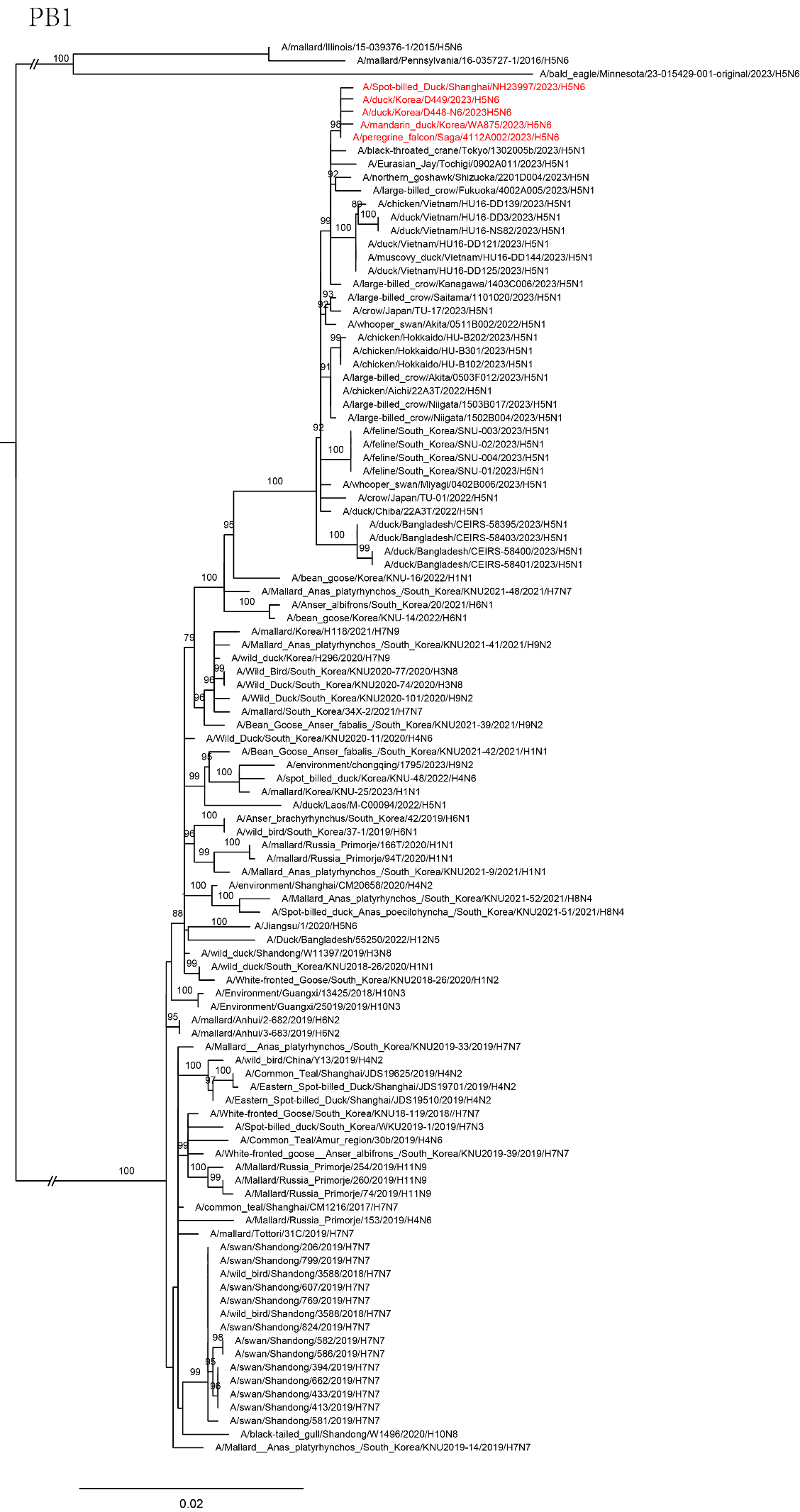


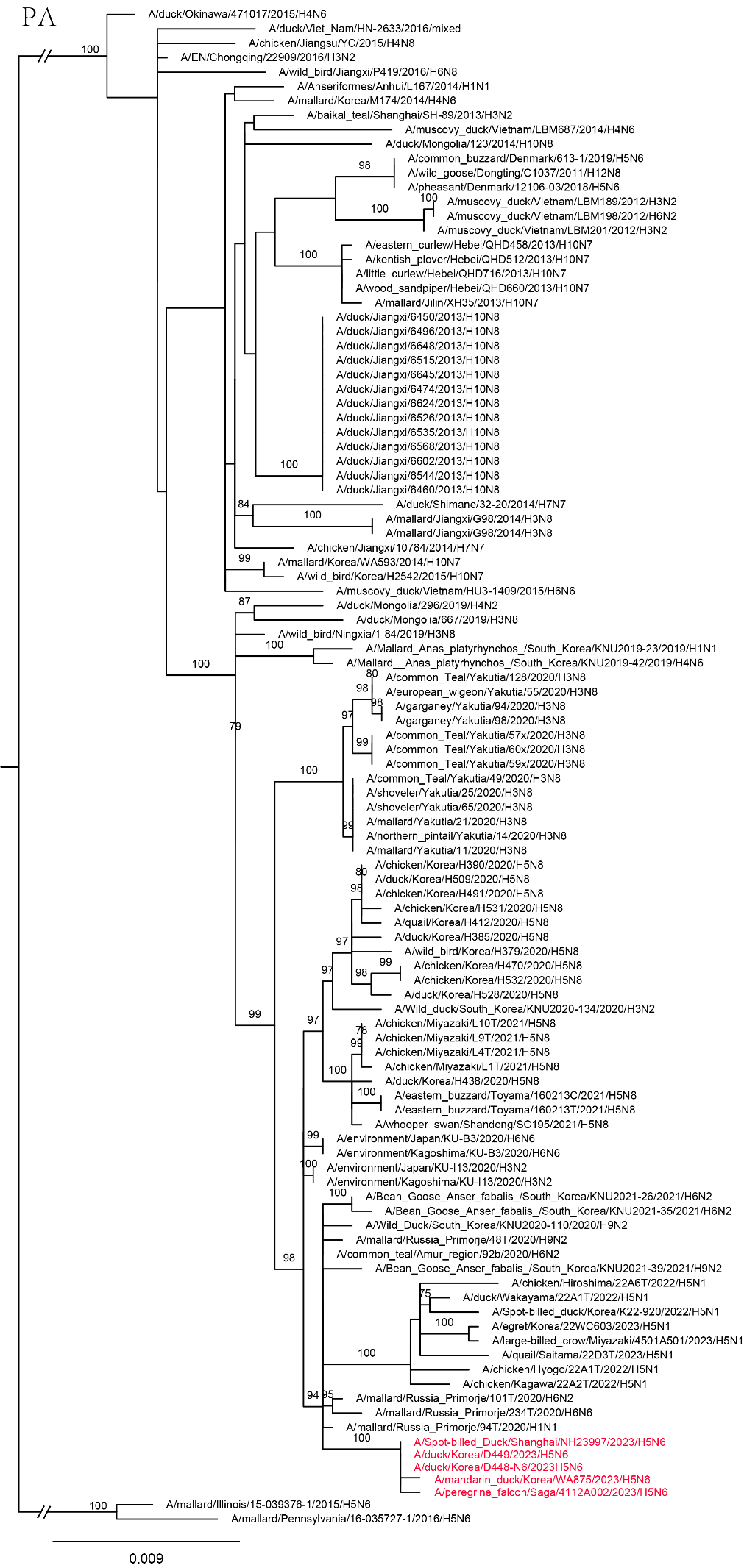


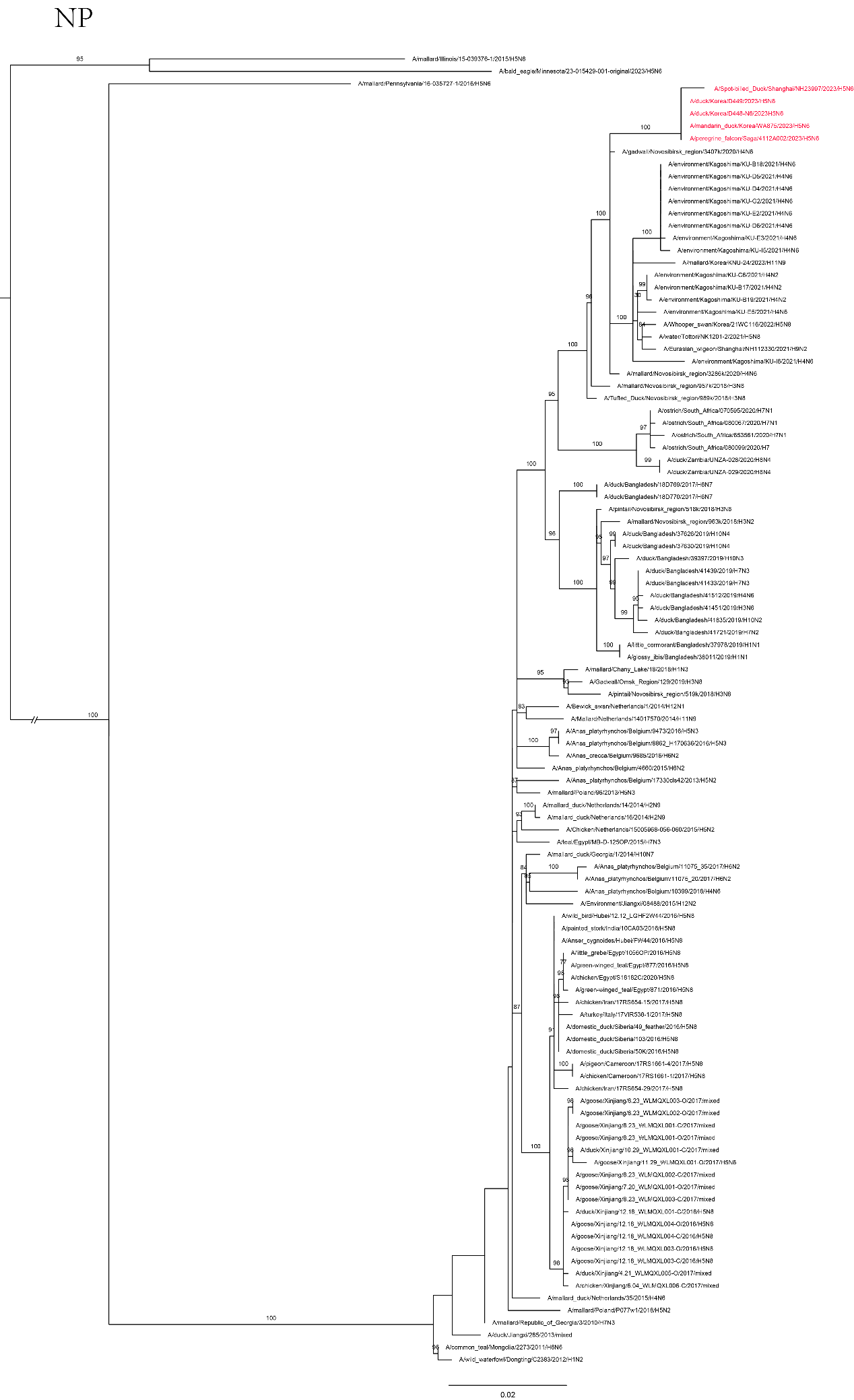


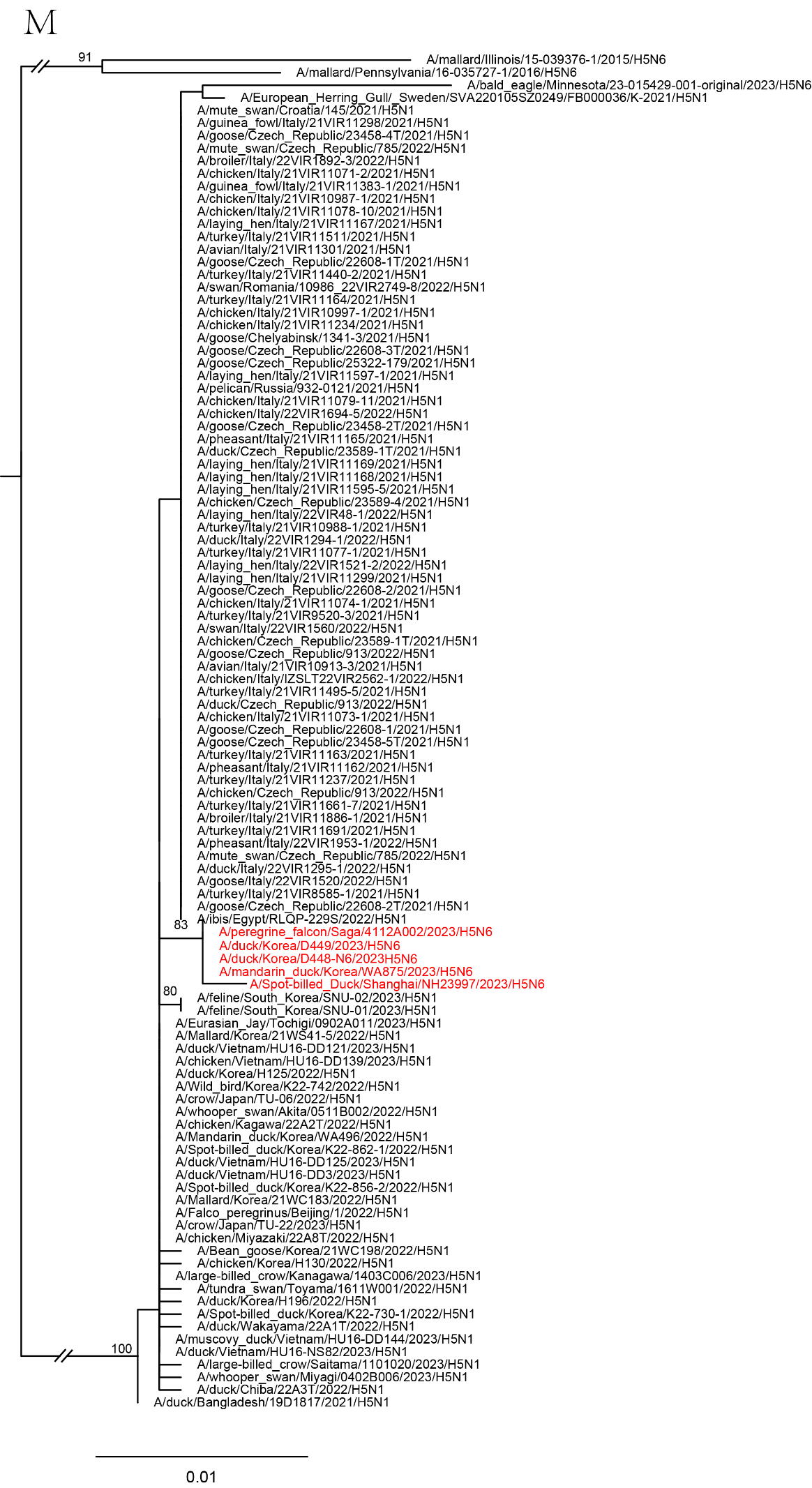


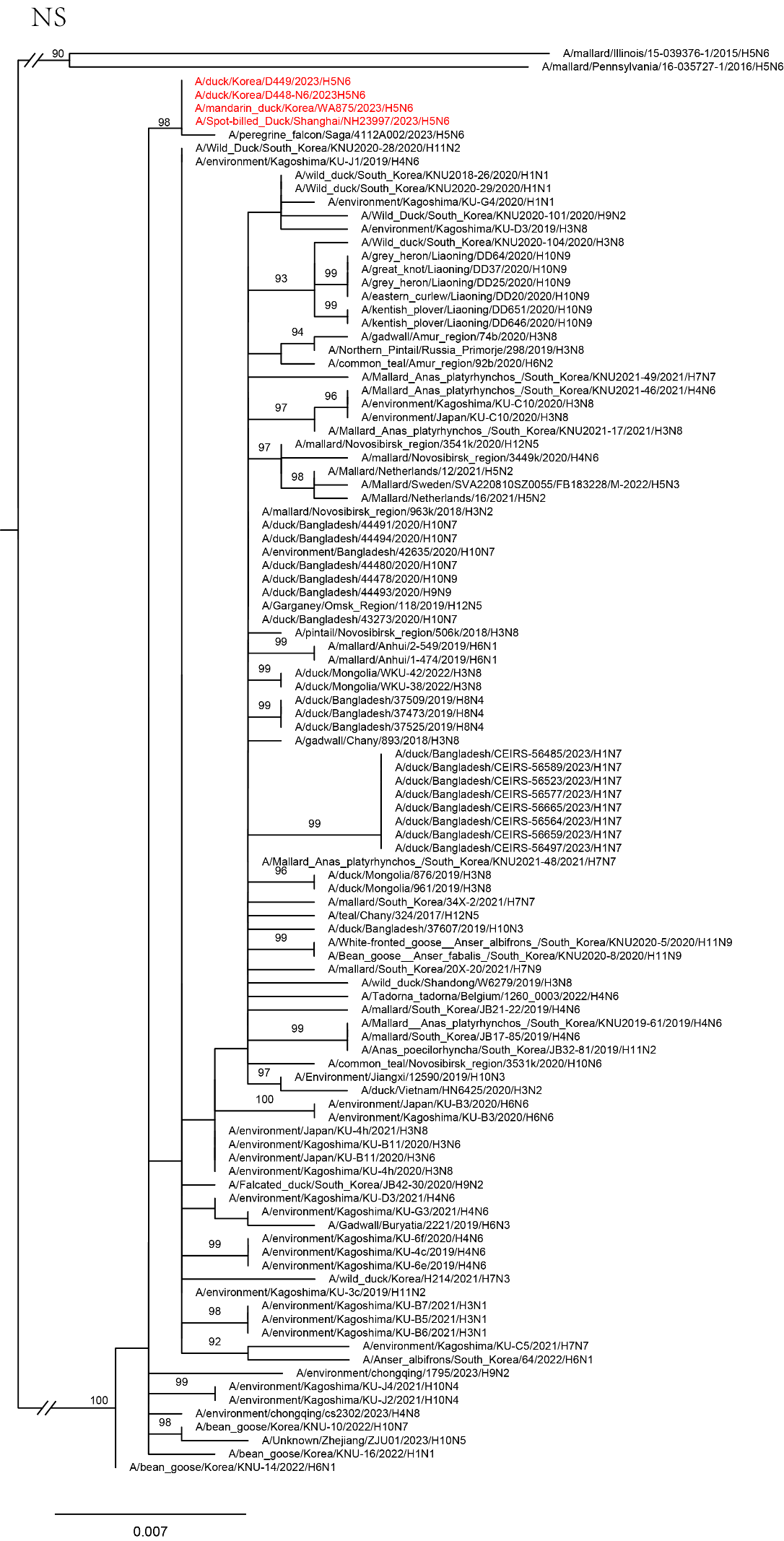


**Figure S1.** Maximum-likelihood trees of six internal genes. (a) PB2, (b) PB1, (c) PA, (d) NP, (e) M, and (f) NS of the 2023-H5N6-like viruses (red) isolated in 2023.
